# Atractylenolide III Attenuates Angiogenesis in Gastric Precancerous Lesions Through the Downregulation of Delta-Like Ligand 4

**DOI:** 10.3389/fphar.2022.797805

**Published:** 2022-06-30

**Authors:** Ying Gao, Jundong Wang, Maoyuan Zhao, Ting Xia, Qingsong Liu, Nianzhi Chen, Wenhao Liao, Zhongzhen Zeng, Fengming You, Jinhao Zeng

**Affiliations:** ^1^ Oncology Department, Hospital of Chengdu University of Traditional Chinese Medicine, Chengdu, China; ^2^ Gastroenterology Department, Hospital of Chengdu University of Traditional Chinese Medicine, Chengdu, China; ^3^ TCM Regulating Metabolic Diseases Key Laboratory of Sichuan Province, Hospital of Chengdu University of Traditional Chinese Medicine, Chengdu, China; ^4^ Geriatric Department, Hospital of Chengdu University of Traditional Chinese Medicine, Chengdu, China

**Keywords:** gastric precancerous lesions, angiogenesis, DLL4, atractylenolide III, microvessel

## Abstract

**Background:** Blocking and even reversing gastric precancerous lesions (GPL) is a key measure to lower the incidence of gastric cancer. Atractylenolide III (AT-III) is a mainly active component of the Atractylodes rhizome and has been widely used in tumor treatment. However, the effects of AT-III on GPL and its mechanisms have not been reported.

**Methods:** H & E staining and AB-PAS staining were employed to evaluate the histopathology in the gastric mucosa. In parallel, CD34 immunostaining was performed for angiogenesis assessment, and transmission electron microscope for microvessel ultrastructural observation. Investigation for the possible mechanism *in vivo* and *in vitro* was conducted using immunohistochemistry, RT-qPCR and western blotting.

**Results:** In most GPL specimens, AT-III treatment reduced microvascular abnormalities and attenuated early angiogenesis, with the regression of most intestinal metaplasia and partial dysplasia. Meanwhile, the expression of VEGF-A and HIF-1α was enhanced in GPL samples of model rats, and their expressions were decreased in AT-III-treated GPL rats. Moreover, DLL4 mRNA and protein expression were higher in GPL rats than in control rats. DLL4 protein expression was significantly enhanced in human GPL tissues. In addition, AT-III treatment could diminish DLL4 mRNA level and protein expression in the MNNG-induced GPL rats. *In vitro* study showed that in AGS and HGC-27 cells, DLL4 mRNA level and protein expression were significantly decreased after AT-III treatment. However, AT-III had no significant regulatory effect on Notch1 and Notch4.

**Conclusion:** AT-III treatment is beneficial in lessening gastric precancerous lesions and attenuating angiogenesis in rats, and that may be contributed by the decrease of angiogenesis-associated HIF-1α and VEGF-A, and downregulation of DLL4.

## Introduction

Gastric precancerous lesions (GPL) refer to the pathological changes of intestinal metaplasia (IM) and dysplasia accompanied by chronic atrophic gastritis, which are positively associated with the incidence of gastric cancer (GC) (Huan et al., 2015). Therefore, it is an effective measure to pay attention to the early diagnosis and treatment of GPL for the secondary prevention of GC. The pathogenesis of GPL is still unclear, and endoscopic mucosal dissection is currently recommended as the main treatment for severe dysplasia and early gastric cancer (Pimentel-Nunes et al., 2019). However, in clinical practice, there is no specific treatment for most GPL patients. Thus, it is essential for us to find new and effective treatments for GPL.

At present, many researchers have focused more attention on natural bioactive components due to their high activity and low cytotoxicity (Ambriz-Pérez et al., 2016; Leyva-López et al., 2016; Villarreal-García et al., 2016). Atractylodes macrocephala is one of the traditional Chinese medicinal herbs, which has obvious curative effects on anorexia, dyspepsia and diarrhea (Wei et al., 2011; Zheng et al., 2012). Atractylenolide III (AT-III) is the main bioactive component of Atractylodes macrocephala and it has been proved to possess pharmacological properties include anti-inflammatory, antioxidative, anti-tumor and anti-angiogenesis effects (Hoang et al., 2012; Wang et al., 2015; Huai and Ding, 2020; Bailly, 2021; Sheng et al., 2021). A previous study demonstrated that AT-III might play a gastroprotective role in ethanol-induced acute gastric ulcer by reducing extracellular matrix damage (Wang et al., 2010). Recently, AT-III has been found to inhibit the proliferation of gastric cancer cells and induce apoptosis of gastric cancer cells, thus playing an anti-tumor role (Ji et al., 2019). In addition, AT-III, as an anti-tumor agent, restricts the recruitment of new blood vessels required for tumor formation and growth by inhibiting angiogenesis (Wang et al., 2015). However, the anti-angiogenesis mechanisms of AT-III in GPL treatment are still unclear.

Angiogenesis, defined as the basic process of forming new blood vessels from pre-existing ones, is closely associated with tumor growth, invasion and metastasis (Judah, 2002; Nienhüser and Schmidt, 2017). Therefore, targeting angiogenesis is the focus of tumor therapy. Hypoxia inducible factor-1α (HIF-1α) is the main drive factor of angiogenesis, and abnormal activation of HIF-1α leads to VEGF overexpression to a large extent, which is critical for angiogenesis (Arany et al., 2008; Tirpe et al., 2019). Vascular endothelial growth factor-A (VEGF-A), also called VEGF, is considered to be a major regulatory factor of tumor angiogenesis, which can stimulate tumor angiogenesis and increase tumor vascular permeability. (Hoeben et al., 2004; Korpanty et al., 2011). More importantly, recent reports have found that DLL4/Notch signaling is the most significant of all the signaling pathways involved in tumor angiogenesis (Yen et al., 2015; McKeage et al., 2018). In humans, four Notch receptors (Notch 1–4) and five ligands (delta-like ligands 1, 3, 4 and Jagged 1 and 2) have already been identified. Among these, Notch1, Notch4, and DLL4 were confirmed to play a pivotal role in angiogenesis (Sainson and Harris, 2008). As the specific ligand for Notch1 and Notch4, DLL4 expression is closely related to tumor angiogenesis and metastasis (Benedito et al., 2009; Li et al., 2011; Miao et al., 2017). Studies have shown that DLL4 overexpression is associated with TNM stage and distant metastasis in GC patients, indicating an association with poor prognosis in GC patients (Ishigami et al., 2013; Du et al., 2014). Although the role of the molecules in promoting angiogenesis in gastric cancer has been reported in recent years, what role they might play in GPL remains unclear.

In the research, the effects of AT-III on GPL angiogenesis and expression of angiogenesis related factors were observed. We hoped to test the hypothesis that the anti-angiogenesis properties of AT-III are related to the regulation of angiogenesis-associated markers HIF-1α and VEGF-A, as well as the DLL4/Notch signaling pathway. Our results may provide experimental evidence for AT-III to inhibit GPL angiogenesis.

## Materials and Methods

### Animals and Ethics Statement

Half male and half female SD rats, weight 180–200 g, were provided by Chengdu Dashuo Experimental Animal Co., Ltd. The rats were fed standard rat chow at room temperature of 22–24°C, relative humidity of 40–60% and light-dark cycle of 12 h. The rats were given adaptive feeding for 1 week before the experiment. All animal procedures are approved and permitted by the Institutional Animal Care and Use Committee (Animals use license: SCXK-2020-030, ethical approval number: 2019-17/24).

### Clinical Tissue Samples

56 cases of GPL gastric mucosa and 46 cases of normal gastric mucosa were collected from the Teaching Hospital of Chengdu University of Traditional Chinese Medicine, and retrospectively analyzed. Formalin fixed and paraffin-embedded tissue samples were stored at room temperature. All specimens were confirmed by pathological examination. This present study was permitted by the Institutional Review Board of the Teaching Hospital of Chengdu University of Traditional Chinese Medicine (Chengdu, China) (approval no. 2018KL-023). Each participant included in the study signed the written informed consent.

### Establishment of Gastric Precancerous Lesions Model in Rats and Drug Administration

**FIGURE 1:**
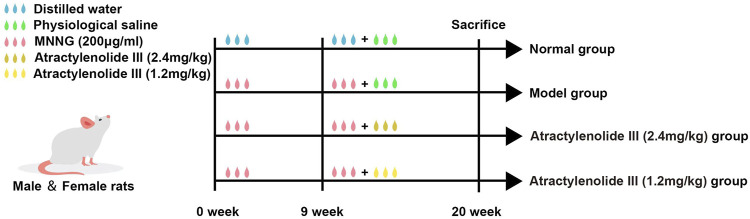
Flowchart of experimental design. 1) Normal group (treated with distilled water and physiological saline, control group); 2) Model group (treated with MNNG and physiological saline); 3) Atractylenolide III (2.4 mg/kg) group (treated with MNNG and high dose AT-III); 4) Atractylenolide III (1.2 mg/kg) group (treated with MNNG and low dose AT-III). MMNG, N-methyl-N′-nitro-N-nitrosoguanidin.

The experiment flow chart was shown in Figure 1. Briefly, SD rats were randomly divided into four experimental groups (*n* = 10 per group): control group (treated with distilled water and physiological saline), model group (treated with MNNG and physiological saline), high-dose Atractylenolide III group (treated with MNNG and AT-III, 2.4 mg/kg/d) and low-dose Atractylenolide III group (treated with MNNG and AT-III, 1.2 mg/kg/d) (cat. no. BZP0374, Hefei Bomei Biotechnology Co., Ltd., China). The GPL rats model was set up based on the literatures (Saito et al., 1970; Tatematsu et al., 1988). To establish the GPL rat model, the rats were given MNNG at 5 ml/kg by gavage once a week and allowed to drink MNNG solution (200 μg/ml) (cat. no. M0527, Tokyo Chemical Industry Co., Ltd., Japan) *ad libitum*, and underwent starvation and satiety conversion every other day. At the end of 9th week, 2 model rats were randomly selected and sacrificed, and then detected for GPL. At the 10th week, rats in the high-dose and low-dose Atractylenolide III groups were given AT-III at 2.4 mg/kg and 1.2 mg/kg by gavage, respectively, while rats in the control group and model group were intragastric with physiological saline (10 ml/kg) for 10 weeks, once a day. At the end of 20th week, all rats were sacrificed with sodium pentobarbital (140 mg/kg i. p.) after 12 h fasting. Following sacrifice by cervical dislocation, stomachs were harvested immediately.

### Cell Culture

Human gastric cancer cell lines (AGS and HGC-27) were obtained from the Centre Laboratory of Affiliated Hospital of Chengdu University of TCM. Cells were divided into 3 groups: control group, high-dose AT-III group (120 µM), low-dose AT-III group (80 µM). Cell lines were cultured in RPMI-1640 medium (Gibco, United States) containing 10% fetal bovine serum (FBS) at 37°C and 5% CO_2_ saturated humidity. CCK-8 assay was used to determine relative cell viability after AT-III treatment for 24 h. The IC_30_ and IC_50_ of AT-III at 24 h in HGC-27 and AGS cells were found to be 80 and 120 μM, respectively.

### Pathological Analysis

Gastric tissues were removed and fixed overnight in 10% neutralized formalin, followed by dehydration with alcohol and xylene. Then, the 3-µm paraffin-embedded sections were prepared and dipped in hematoxylin and eosin (H & E) using standard protocols. According to the manufacturer’ sintroductions, the types of intestinal metaplasia were examined by Alcian Blue-Periodic Acid Schiff (AB-PAS staining). The paraffin sections were deparaffinized to water. The sections were stained with Alcian Blue staining solution for 5–10 min. The slices were oxidized with 1.0% periodic acid solution for 10 min, rinsed in running water for several minutes, and washed twice in distilled water. The sections were stained with Schiff solution for 15–30 min without light, rinsed with running water for 5–10 min. After the slices were dried, the nucleus was lightly stained with Mayer hematoxylin for about 1 min, rinsed with running water for several minutes, dehydrated with gradient alcohol, transparented with xylene and sealed with neutral gum. Neutral mucins in normal mucosa were stained magenta, while acidic mucins in IM lesion were stained blue. The morphological changes of the sections were observed by light microscope (IX71; Olympus Corporation) and the incidence of GPL in the rats were analyzed (Tytgat, 1991; Riddell, 1995). The magnifications used were ×100 and ×200.

### Evaluation of Microvessel Density

The expression of CD34 was detected by immunohistochemical staining (IHC) to assess microvessel density (MVD) in gastric mucosa. In order to measure MVD, we used the method described by Weidner to perform the quantitative vessel counts (Vermeulen et al., 1996). To be specific, the tissue sections were scanned at low-power magnification (×40 and ×100) to identify areas with the highest angiogenesis (also known as hot-spot). Then, counting the stained microvessels in 3 random views of the “hot-spot” area at high-power (×200). The microvascular density value was figured as the mean value of the 3 field counts (×200).

### Transmission Electron Microscopy

The ultrastructure of the gastric mucosa was observed using TEM. The gastric mucosa tissue specimens (1 mm^3^) were fixed in 2.5% glutaraldehyde prepared in phosphate buffer for 2.5 h, and re-fixed in 1% osmium tetroxide in phosphate buffer for 2 h. The tissues were washed with buffer, dehydrated in gradual ethanol, then dipped twice in a mixture of acetone and epoxy resin, and embedded in capsules filled with epoxy resin, heated overnight at 70°C, and 70-nm ultrathin sections were sliced with LKB microtome. Images were observed and imaged by TEM (H-7650; Hitachi Ltd.) and used to describe the ultrastructure of microvessel. The magnification was ×10,000.

### Immunohistochemical Staining

Gastric sections were embedded in paraffin and cut into 3 µm slices for IHC assay. The sections were heated at 97°C for 20 min, soaked in 3% hydrogen peroxide solution for 15 min and blocked with 5% bovine serum albumin for 30 min. The sections were incubated overnight at 4°C with primary antibodies against CD34 (lot ZDP0112111, R & D Systems, United States), VEGF-A (lot GR116031-1, Abcam, United Kingdom), HIF-1α (lot L1212, Santa Cruz Biotechnology, United States), Notch1 (ab52301, Abcam, United Kingdom), Notch4 (ab184742, Abcam, United Kingdom), and DLL4 (ab7280, Abcam, United Kingdom). Then, the sections were stained with diaminobenzidine and counterstained with hematoxylin to detect the results. Three fields were randomly selected under the light microscope for photographing. Quantification of expression levels was determined by mean of integrated optical density and analyzed by Image Pro Plus 6.0 software (Media Cybernetics, Inc.). The magnification used was ×200.

### Western Blot

Total protein was extracted with Radioimmunoprecipitation assay (RIPA) lysis buffer containing protease inhibitors, and the protein concentration was measured by the BCA assay. Equal quantities of the total protein were loaded into wells and separated using 10% SDS-PAGE, and then transferred electrophoretically to polyvinylidene fluoride membranes. After blocking in 5% nonfat dry milk for 2 h, the membranes were incubated overnight with primary antibodies at 4°C. The dilution ratios of primary antibodies in the experiment were as follows: DLL4 (1: 1000), Notch1 (1: 1000), Notch4 (1: 1000), and GAPDH (1: 1000) (lot 00093663, Proteintech Co., Ltd., United States). Next, the membranes were washed with PBST, then the secondary antibodies were added and incubated at 37°C for 1 h. The gray values of the bands were quantified and normalized to GAPDH by the Image-Pro Plus software version 6.0 (Media Cybernetics, Inc.).

### Real-Time Quantitative RT-PCR

TRIzol kit (G3013; Servicebio) was used to extract total RNA from tissues and cells. The RT-qPCR was performed with SYBR Green qPCR Mix kit and 7500 Fast Real-Time PCR System (Applied Biosystems Inc.) to detect the mRNA levels of Notch1, Notch4, and DLL4. The differences of amplification were calculated by the 2^−ΔΔCt^ method. The primer sequences used in rats were as follows: *DLL4* forward 5′-TGC​CAC​TTC​GGT​TAC​AC-3′ and reverse 5′-TGA​CAC​ATT​CGT​TCC​TCT​C-3′; *Notch1* forward 5′-AGC​CAG​TAA​GCC​AAG​T-3′ and reverse 5′-ACA​GTC​CAT​CCT​CAG​TT-3′; *Notch4* forward 5′-CAG​CCC​GAG​CAG​ATG​TAG​GA-3′ and reverse 5′-CGG​CGT​CTG​CTC​CCT​ACT​GT-3′; *18S* 5′-ACG​GCT​ACC​ACA​TCC-3′ and reverse 5′-CAG​ACT​TGC​CCT​CCA-3′. The human DLL4 primer was Cat#HQP013577 (Hs-QRP-20948 DLL4; GeneCopoeia, Inc) and GAPDH primer was Cat#HQP006940 (Hs-QRP-20169 GAPDH; GeneCopoeia, Inc).

### Statistical Analysis

The data analyses were performed by SPSS 23.0 software (IBM Inc.). All quantitative data were presented as mean ± SEM. One-way ANOVA was used to evaluate the differences between groups, Tukey method was used for homogeneous data and Dunnett’s T3 method was used for non-homogeneous data. Unpaired Student’s t-test was used to analyze the differences in DLL4, notch1 and notch4 expressions between GPL group and normal group. Pearson’s χ^2^ test and Fisher’s exact test were performed to assess DLL4, notch1, and notch4 expression and clinicopathological characteristics. *p* < 0.05 was considered statistically significant.

## Results

### Atractylenolide III Improves the General Condition and Alleviates Histopathological Changes of the Gastric Mucosa in Gastric Precancerous Lesions Rats

**FIGURE 2:**
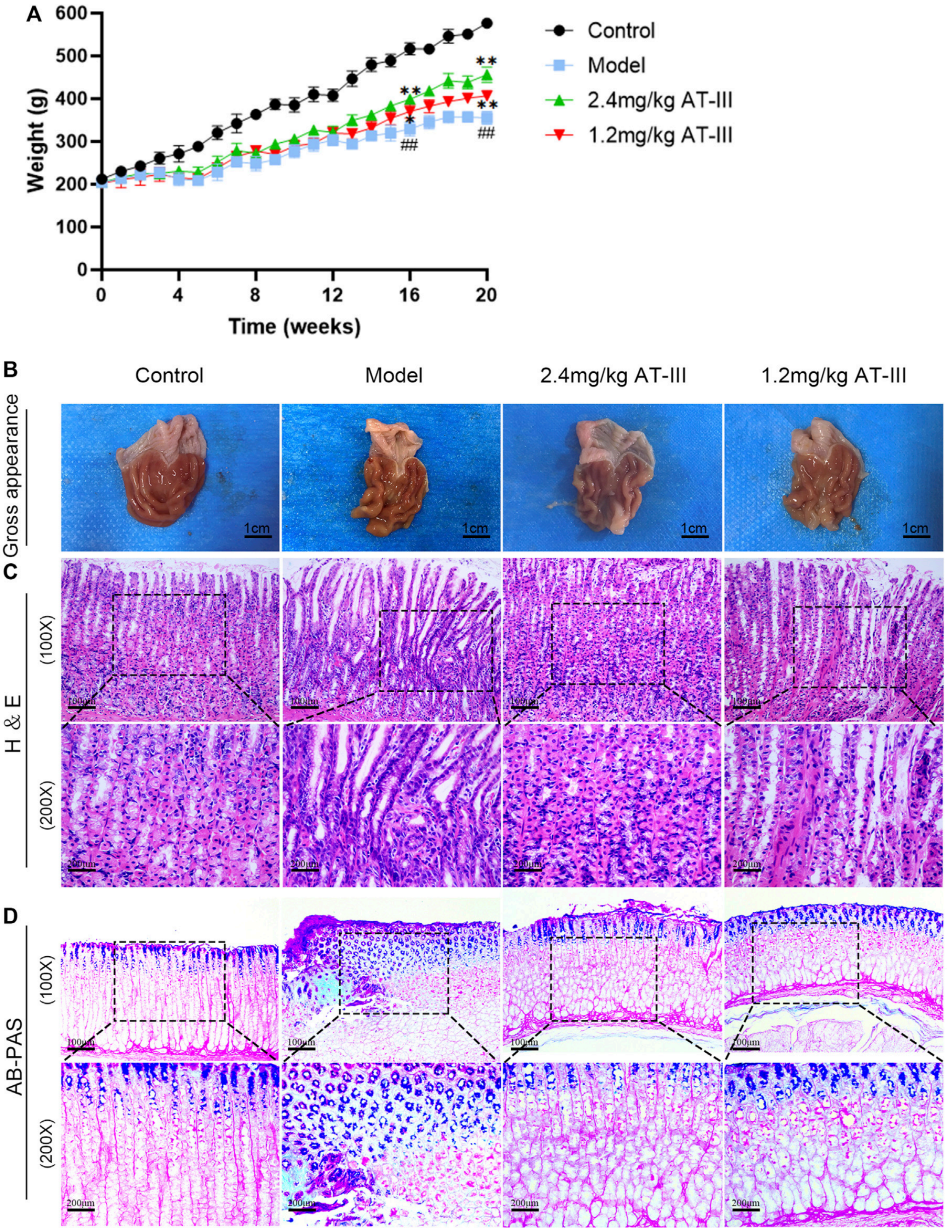
Effects of AT-III on pathomorphology of dysplasia, gastric intestinal metaplasia and body weight in GPL rats. **(A)** Effect of AT-III treatment on body weight of GPL rats. **(B)** Gross evaluation of the gastric mucosa. **(C)** Representative images of H & E staining of the gastric epithelium (×100, ×200). **(D)** Evaluation for intestinal metaplasia using AB-PAS staining (×100, ×200). ^##^ *p* < 0.01 versus the control group; ^*^ *p* < 0.05, ^**^ *p* < 0.01 versus the model group. Abbreviations: AT-III, Atractylenolide III; GPL, gastric precancerous lesions; H & E, hematoxylin and eosin; AB-PAS, alcian blue-periodic acid schiff.

The control rats appeared relaxed, moved quickly, ate well, and had hard, grainy feces. In contrast, the model rats seemed less energetic, moved slowly, ate less and had diarrhea. The body weight of the model rats decreased remarkably compared with the control group (*p* < 0.01). AT-III administration (concentration of 1.2, 2.4 mg/kg) could partly preserve the body weight of rats (*p* < 0.05; *p* < 0.01; Figure 2A). These results suggest that AT-III prevents GPL-associated body weight loss.

We used H & E staining to assess the histopathological changes of gastric mucosa. Our data indicated that there was no intestinal metaplasia or dysplasia in the control group, and the difference was statistically significant compared with the model group (*p* < 0.01). The incidence of GPL in the model group, AT-III administration groups (1.2 mg/kg, 2.4 mg/kg) was 90% (9/10), 50% (5/10), and 30% (3/10), respectively. There were significant differences between the AT-III group (2.4 mg/kg) and the model group (*p* < 0.05). As shown in Table 1. Morphologically, rats in the control group exhibited normal macroscopic appearance of gastric mucosa. The glands and structure of gastric epithelial cells were normal, and there was little or no inflammatory infiltration in gastric epithelium under light microscope. In contrast, the gastric mucosa of the model rats appeared as dark red, with poor lustrousness, little mucus and rough surface. Light microscope revealed the arrangement of gastric mucosa glands was irregular and crowded and back-to-back tubular structure. In addition, the gastric epithelial cells showed enlarged and hyperchromatic nuclei, increased nuclear-cytoplasmic ratio, loss of nuclear polarity, and gastric mucosa stimulated cavity fusion, suggesting dysplasia lesion of the gastric mucosa. As expected, atypical hyperplasia and inflammatory infiltration were inhibited to varying degrees in most rats treated with AT-III (Figures 2B,C). These observations suggested that AT-III can inhibit or even reverse the process of dysplasia and protect gastric mucosa.

**TABLE 1:** The incidence rate of GPL in each group.

Group	Number	Intestinal metaplasia			Dysplasia			GPL incidence (%)
		Mild	Moderate	Severe	Mild	Moderate	Severe	
Control group	10	0	0	0	0	0	0	0
Model group	10	1	2	1	1	1	3	90
1.2 mg/kg AT-III group	10	2	1	0	0	1	1	50
2.4 mg/kg AT-III group	10	1	1	0	1	0	0	30

The degree of intestinal metaplasia was evaluated using AB-PAS staining. In the control rats, the neutral mucins in gastric mucosa were stained red, indicating no intestinal metaplasia. In the model group, gastric mucosa with lesion of intestinal metaplasia were stained blue or purple. After AT-III intervention, we found that intestinal metaplasia was visibly regressed as compared with that in the model rats (Figure 2D). Our observation showed that AT-III could effectively reverse gastric intestinal metaplasia in GPL rats.

### Atractylenolide III Ameliorates Microvascular Abnormalities

**FIGURE 3:**
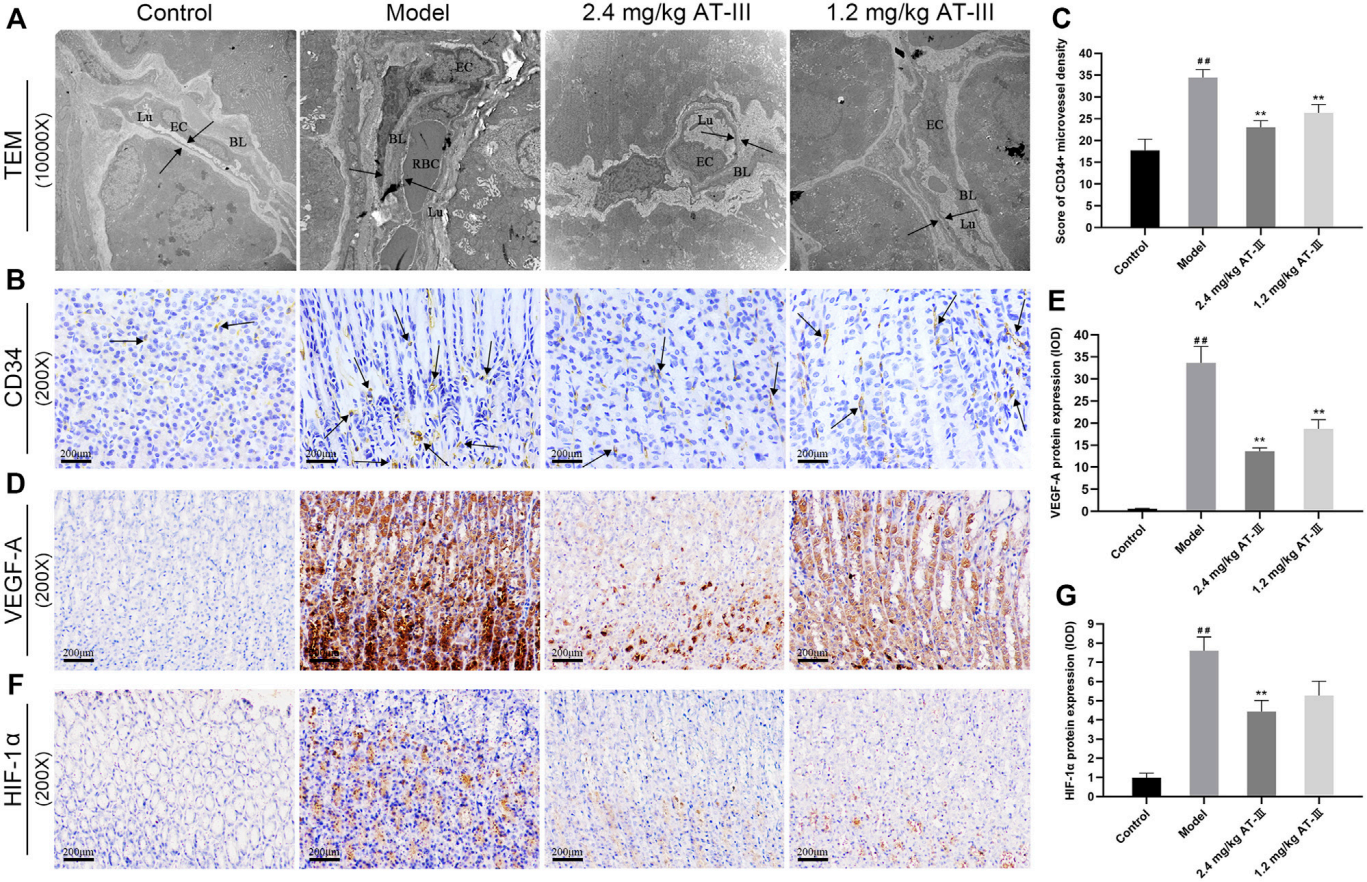
Evaluation of microvessel density in gastric mucosa. **(A)** Representative images of ultrastructures of epithelial cells using TEM (×10000). **(B)** CD34-labelled microvessels in gastric mucosa of each group (IHC, ×200). Expression of VEGF-A **(D)** and HIF-1α **(F)** protein in different gastric mucosa (IHC, ×200). Effect of AT-III on CD34 levels **(C)**, VEGF-A **(E)** and HIF-1α **(G)** protein expressions in gastric mucosa from various groups (*n* = 10). Data are presented as mean ± SEM. RBC, red blood cell; EC, endothelial cell; BL, basal lamina; Lu, lumen. ^##^ *p* < 0.01 versus the control group; ^**^ *p* < 0.01 versus the model group. RBC, red blood cell; EC, endothelial cell; BL, basal lamina; Lu, lumen; TEM, transmission electron microscopy; AT-III, Atractylenolide III; IHC, immunohistochemistry; SEM, standard error of mean.

The morphological changes of microvessels in gastric mucosa were observed under TEM. The vascular inner diameter of microvessels in the control group was normal, the basal lamina was smooth and the structure was clear, complete and continuous with uniform thickness and homogeneous electron density. In contrast, we found that the microvascular lumen was dilated, the vascular inner diameter was significantly reduced, the basal lamina was thickened and rough, and the basement membrane was irregular and discontinuous in model rats. Partial or complete occlusion of some vascular lumens by erythrocytes was also observed. In the low-dose AT-III group (1.2 mg/kg), vascular lumen inner diameter of rats was mild to moderately reduced, the basal lamina surface was still rough, and part of the basal lamina was broken and discontinuous. Furthermore, in the high-dose AT-III group (2.4 mg/kg), the capillary wall of rats was relatively smooth, the basal lamina is slightly fractured and discontinuous without obvious thickening, the inner diameter of vascular lumen was slightly decreased. Most of the nuclear membrane is clear and intact, and the distribution of nuclear chromatin is normal (Figure 3A). Therefore, AT-III intervention showed a potent protective effect on microvascular abnormalities in GPL rats.

### Atractylenolide III Reduces the CD34-Labled Microvessel Density and Decreases VEGF-A and HIF-1α Protein Expressions

The expression of the angiogenesis marker CD34 in GPL tissues was detected by immunohistochemistry to analyze the effect of AT-III on angiogenesis. The number of CD34^+^ microvessels was increased in most GPL tissues, suggesting active angiogenesis, whereas these microvessels were sparse in control tissues. Furthermore, we noticed that more GPL rats with dysplasia had a larger number of microvessels than IMs, and severe dysplasia had more microvessels than mild or moderate dysplasia. In contrast, we noted a significant reduction in CD34^+^ microvascular counts in many AT-III-treated rats. These results demonstrated that CD34^+^ microvessel density levels were significantly increased in model rats compared to the control group. But it decreased markedly after AT-III intervention, indicating that AT-III effectively reduced angiogenesis in GPL rats (Figures 3B,C).

IHC was used to evaluate whether VEGF-A and HIF-1α inhibition was associated with anti-angiogenic ability of AT-III. The data confirmed that HIF-1α and VEGF-A were sparsely expressed in normal gastric mucosa, while HIF-1α and VEGF-A protein expression were increased in GPL rats. As expected, the expressions of HIF-1α and VEGF-A protein in gastric mucosa were significantly decreased after AT-III intervention, and the difference was statistically significant (*p* < 0.01), but AT-III had no significant inhibitory effect on HIF-1α protein at a concentration of 1.2 mg/kg (*p* > 0.05). Interestingly, we observed that the reduction of HIF-1α and VEGF-A in GPL tissues was often accompanied by the attenuation of CD34^+^ expression, suggesting that inhibition of HIF-1α and VEGF-A might be beneficial in AT-III-alleviated angiogenesis (Figures 3D–G).

### DLL4 is Over-Expressed in Human Samples of Gastric Precancerous Lesions

**FIGURE 4:**
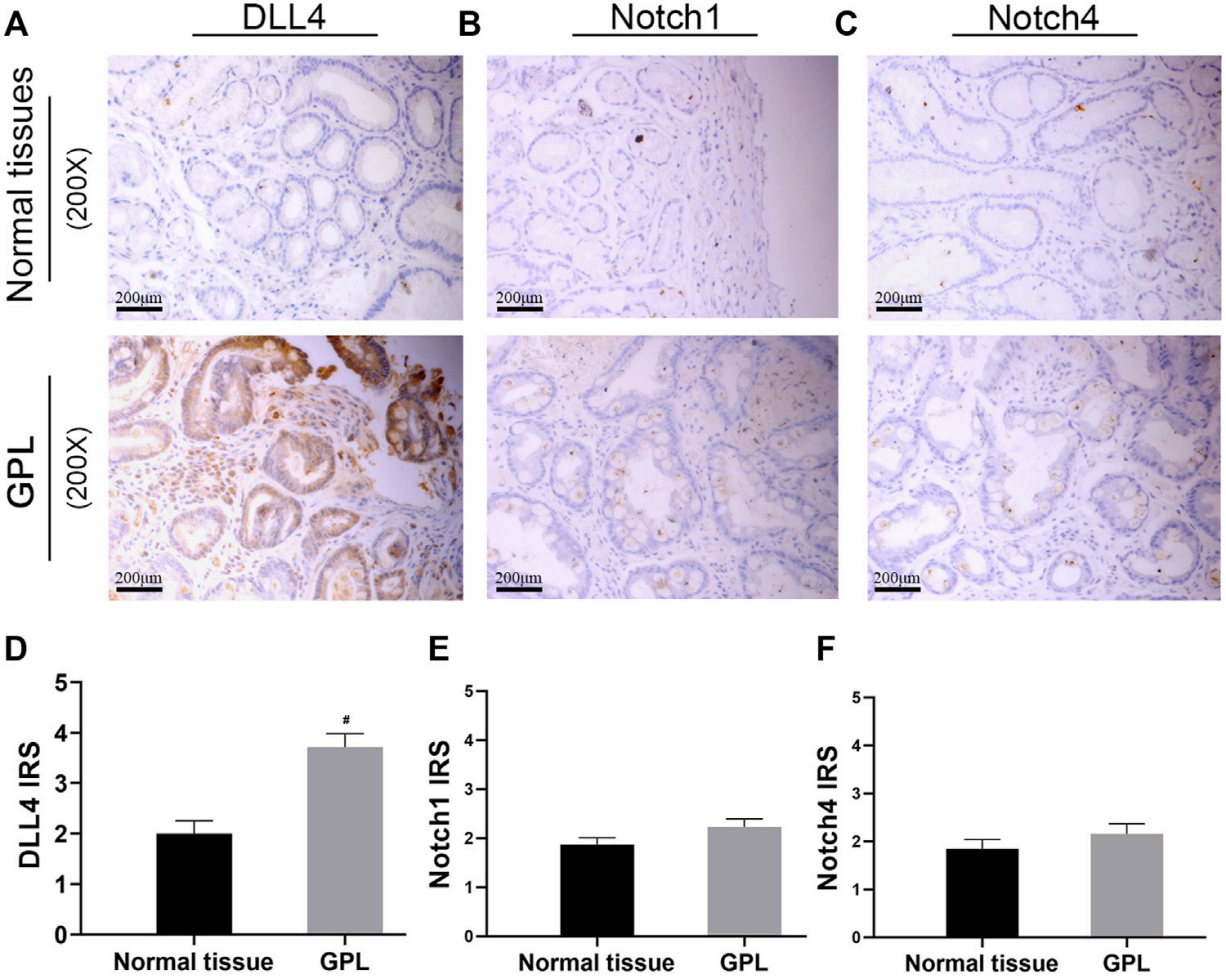
DLL4 mRNA expression is upregulated in gastric cancer and DLL4 protein is over-expressed in human GPL specimens. Representative IHC images demonstrating the expression of DLL4 **(A)**, Notch1 **(B)** and Notch4 **(C)** in GPL and normal tissues (×200). Semi-quantitative analysis of DLL4 **(D)**, Notch1 **(E)** and Notch4 **(F)** protein expression in human specimens (*n* = 102). ^#^ *p* < 0.05 versus the control group. Data are presented as mean ± SEM. Abbreviations: AT-III, Atractylenolide III; GPL, gastric precancerous lesions; GC, gastric cancer; IHC, immunohistochemistry; SEM, standard error of mean.

In order to verify the high expression of Notch1, Notch4, and DLL4 in GPL, we investigate the expression of Notch1, Notch4, and DLL4 in 56 human GPL specimens and 46 normal specimens. We observed high DLL4 expression in 62.5% (35/56) of the GPL specimens and 41.3% (19/46) of the normal specimens by IHC. At the same sites, we found strong Notch1 expression in 7.1% (4/56) of the GPL specimens and 4.3% (2/46) of the normal specimens. In addition, we observed high Notch4 levels in 14.3% (8/56) of the GPL specimens and 6.5% (3/46) of the normal specimens. We noticed that the DLL4 immunoreactivity was notably stronger in the human GPL specimens than in the healthy controls (Figures 4A,D), while Notch1 and Notch4 were not overexpressed in human samples of GPL (Figures 4B,C,E,F). This evidence supports the association of DLL4 expression with increased angiogenesis in GPL. DLL4 expression was significantly correlated with advanced GPL pathology but not age, gender, location of lesion and Hp infection (Table 2).

**TABLE 2:** Correlation between DLL4 positivity and clinicopathological characteristics.

	Notch1		Notch4		DLL4	
	Low	High	Low	High	Low	High
Age						
<60	33	3	31	5	12	24
≥60	19	1	17	3	9	11
*p* value	0.643		0.909		0.388	
Gender						
Male	33	3	31	5	7	18
Female	19	1	17	3	14	17
*p* value	0.643		0.909		0.187	
Location of lesion						
Body	10	0	9	1	6	4
Angle	10	1	10	1	4	7
Antrum	26	1	21	6	9	18
Multiple	6	2	8	0	2	6
*p* value	0.160		0.374		0.407	
Hp infection						
Negative	22	0	16	6	12	10
Positive	30	4	32	2	9	25
*p* value	0.095		0.025		0.034	
Histopathological category						
Normal gastric epithelium	44	2	43	3	27	19
Gastric precancerous lesions	52	4	48	8	21	35
*p* value	0.551		0.208		0.033	
Intestinal-type metaplasia	24	2	24	2	8	18
*p* value	0.882		0.189		0.333	
High-grade intraepithelial neoplasia	12	1	9	4	5	8
Low-grade intraepithelial neoplasia	16	1	15	2	8	9
*p* value	0.844		0.197		0.638	

### Atractylenolide III Diminishes DLL4 Protein Expression and mRNA Level in Gastric Precancerous Lesions Rats

**FIGURE 5:**
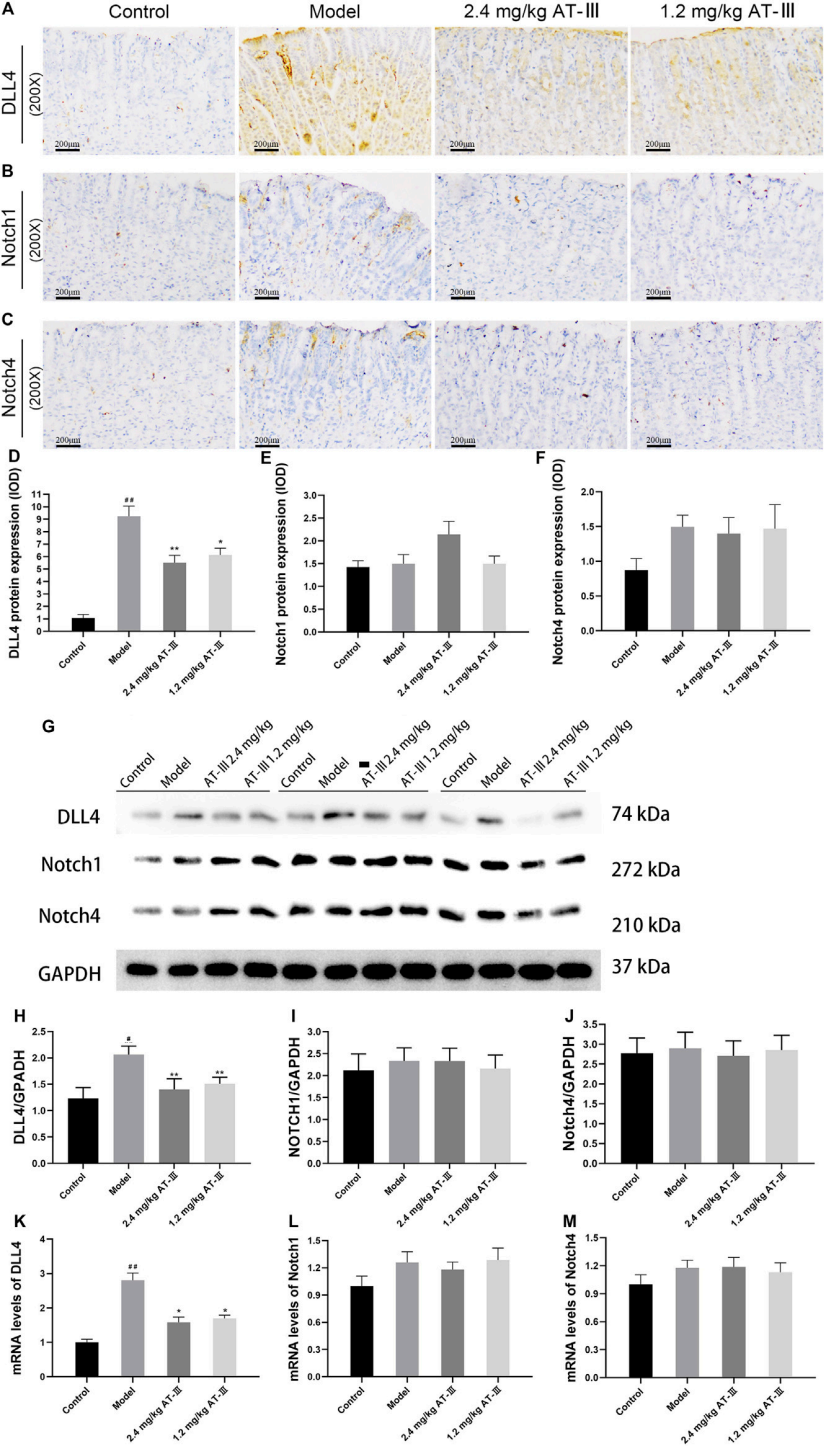
The rat samples were detected by IHC. Expression of DLL4 **(A)**, Notch1 **(B)** and Notch4 **(C)** in gastric mucosa from various groups. Semi-quantitative analysis of DLL4 **(D)**, Notch1 **(E)** and Notch4 **(F)** protein expression levels in each group (*n* = 10). **(G)** Representative images of western blot proteins bands. Quantitative analysis of DLL4 **(H)**, Notch1 **(I)** and Notch4 **(J)** in western blotting bands (*n* = 9). Quantization for mRNA levels of DLL4 **(K)**, Notch1 **(L)** and Notch4 **(M)** in gastric mucosa from various groups (*n* = 6). **(J)** Representative western blotting bands of DLL4, Notch1 and Notch4. ^#^ *p* < 0.05, ^##^ *p* < 0.01 versus the control group; ^*^ *p* < 0.05, ^**^ *p* < 0.01 versus the model group. Data are presented as mean ± SEM. AT-III, Atractylenolide III; IHC, immunohistochemistry; SEM, standard error of mean.

We further examined the expression levels of Notch1, Notch4 and DLL4 in GPL rats to determine the possible mechanism of GPL angiogenesis. The expression of DLL4 protein in gastric mucosa was observed by immunohistochemistry and analyzed by Western blotting. As shown in Figures 5A–C, normal gastric mucosa did not or barely express DLL4, while diffuse and intense cytoplasmic labeling, found in most cases of GPL rats, could be markedly diminished by AT-III. Statistically, GPL rats showed an increased DLL4 protein expression compared with the control group (*p* < 0.01), while AT-III intervention reduced the over-expression. Furthermore, we found that AT-III had a stronger inhibitory effect at a concentration of 2.4 mg/kg on DLL4 over-expression (*p* < 0.01). However, AT-III may have little effect on Notch1 and Notch4 expression (without statistical significance) (Figures 5D–F).

Quantitative analysis of protein expression proved that DLL4 protein expression was elevated in GPL rats compared with control group (*p* < 0.05), whereas AT-III treatment reduced the over-expression (*p* < 0.01) and did not significantly inhibit Notch1 and Notch4 (*p* > 0.05). (Figures 5G–J). Furthermore, RT-qPCR analysis confirmed that DLL4 mRNA level in GPL rats was significantly higher than that in controls (*p* < 0.01). After AT-III intervention, DLL4 mRNA level of rats was dramatically reduced (*p* < 0.05). The data suggested that AT-III could efficiently inhibit DLL4 mRNA level in model rats, but Notch1 and Notch4 mRNA levels were not significantly decreased (Figures 5K–M).

### Atractylenolide III Down-Regulates DLL4 Protein Expression and mRNA Level in Human Gastric Cancer Cell Lines

**FIGURE 6:**
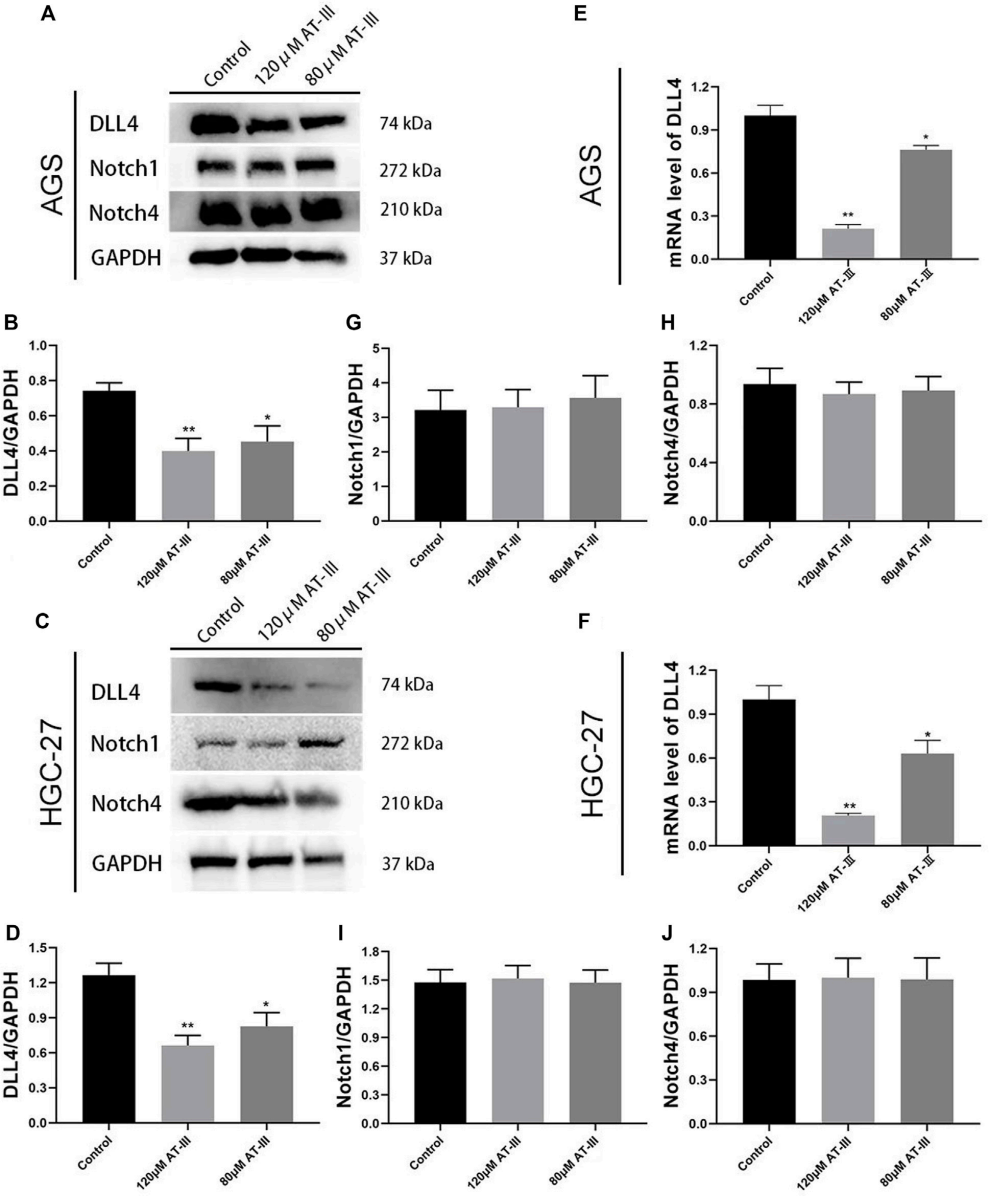
Representative western blotting bands of DLL4, Notch1 and Notch4 in transfected AGS **(A)** and HGC-27 **(C)** cells. Quantitative analysis of DLL4 **(B,D)**, Notch1 **(G,I)**, and Notch4 **(H,J)** in western blotting bands (*n* = 9). RT-qPCR detection for DLL4 mRNA levels in transfected AGS **(E)** and HGC-27 **(F)** cells (*n* = 3). ^*^ *p* < 0.05, ^**^ *p* < 0.01 versus the control group. Data are presented as mean ± SEM. AT-III, Atractylenolide III; SEM, standard error of mean.

DLL4 protein expression was measured by western blotting in human gastric cancer cell lines. The results revealed that AT-III treatment (concentration of 80, 120 µM) down-regulated DLL4 protein expression in AGS and HGC-27 cell lines (*p* < 0.05; *p* < 0.01). Furthermore, AT-III showed a better inhibitory effect at a concentration of 120 µM (*p* < 0.01). And the difference in the gray value was statistically significant (*p* < 0.01; Figures 6A–D).

RT-qPCR detection for DLL4 gene expression in AGS and HGC-27 cells. Our results indicated that the mRNA expression of DLL4 was obviously decreased in the AT-III group (80 µM) compared with control group (*p* < 0.05). The expression of DLL4 gene was further decreased in the AT-III group (120 µM) (*p* < 0.01; Figures 6E,F). All these results clearly indicated that DLL4 protein expression and mRNA level was inhibited after AT-III treatment in AGS and HGC-27 cell lines. However, AT-III treatment did not significantly inhibit the protein expression of Notch1 and Notch4 (*p* > 0.05; Figures 6G–J).

## Discussion

It is well known that GPL is the key stage in the progression of GC. Therefore, early intervention for GPL is important for reducing the morbidity of GC (Huang et al., 2015; Malik et al., 2017). AT-III is a Chinese traditional herb with a long medicinal history isolated from the dried rhizome of Atractylodes macrocephala. In recent years, many basic and clinical studies have shown that AT-III has anti-cancer effects (Ji et al., 2019; Bailly, 2021; Sheng et al., 2021). However, the effects of AT-III on GPL has not been reported. In the research, we observed that AT-III administration could alleviate IM and partial dysplasia in GPL rats, suggesting that AT-III is beneficial in protecting against gastric precancerous lesions.

Folkman’s tumor angiogenesis theory is critical to the treatment of malignant tumors and precancerous lesions (Folkman, 1971). Therefore, inhibiting angiogenesis may be an attractive strategy to prevent and treat malignant transformation of gastric mucosa, which can reduce the morbidity of GC patients. In this research, we found that CD34^+^ MVD in GPL rats was remarkably higher than that in the normal gastric mucosa, which supported the hypothesis of angiogenesis in GPL rats. More importantly, the gastric mucosa often showed a higher CD34^+^ microvessel count in more severe lesions. There was more microvessels in dysplasia than in intestinal metaplasia, and more microvessels in severe dysplasia than in mild or moderate dysplasia. After administration of AT-III, the angiogenesis marker CD34 was significantly decreased in GPL tissues.

HIF-1α and VEGF are considered to be classic factors controlling multiple proangiogenic processes in hypoxic tumors (Rey et al., 2017). A study focusing on the correlation between VEGF and the degree of progression of GPL found that VEGF was overexpressed in GPL, and its expression increased with the severity of gastric mucosal gland atrophy and intestinal metaplasia (Zhao et al., 2019). Experimental evidence suggests that HIF-1α was activated in the early stages of GC. The over-expression of HIF-1α was positively correlated with tumor infiltration depth, MVD and VEGF expression in gastric cancer, and patients with HIF-1α (+)/VEGF (+) had a relatively poor prognosis (Fu et al., 2019). It is worth mentioning that early angiogenesis found in GPL tissues is usually accompanied by activation of HIF-1α and VEGF-A. And we noted that AT-III could inhibit VEGF-A expression in GPL tissues at a concentration of 1.2 mg/kg and 2.4 mg/kg, and reduce HIF-1α expression at a concentration of 2.4 mg/kg.

Recent reports have shown that DLL4/Notch signaling pathway occupies an important role in angiogenesis, including Notch1, Notch4, and DLL4 as key targets (Kangsamaksin et al., 2014). Studies have confirmed that DLL4 expression is up-regulated in the tumor vasculature compared with normal vessels (Schadler et al., 2010; Liu et al., 2015). For example, the expression of DLL4 in clear cell renal tumor vessels was higher than that in normal renal tissues and related to vascular maturation (Huang et al., 2013). Furthermore, studies have indicated that the activation of Notch1, Notch4, and DLL4 is crucial in the initiation and progression of gastric cancer (Du et al., 2014; Qian et al., 2015; Huang et al., 2019). However, the possible role of the molecules in GPL remains unclear.

A previous study demonstrated that AT-III could inhibit breast tumor angiogenesis *in vitro* and *in vivo* (Wang et al., 2015). The possible mechanism is that AT-III suppressed Runx2 activation in endothelial cells, which contributed to the inhibition of MMPs and VEGF-VEGFR2 signaling as well as the anti-angiogenic properties of AT-III (Wang et al., 2015). In our research, DLL4 protein expression were up-regulated in human GPL specimens compared with normal specimens. We found similar results in the animal study, and different concentrations of AT-III treatment could significantly reduce the gene and protein expressions of DLL4. Furthermore, our cell experiments showed that DLL4 protein and gene expressions were markedly down-regulated after AT-III treatment in AGS and HGC-27 cells. The results suggested the role of DLL4 in angiogenesis and provide new ideas for anti-angiogenesis therapy of GPL.

More importantly, our animal and cell experiments showed that AT-III had a stronger inhibitory effect on DLL4 over-expression at a concentration of 2.4 mg/kg and 120 μM, which may be more ideal for GPL intervention, but we need to expand the sample size in the future to further verify our speculation. However, AT-III intervention showed no inhibitory effects on Notch1 and Notch4 expression. The possible reason is that Notch1 and Notch4 may not be the targets for AT-III treatment of GPL. Furthermore, the superiority of AT-III in GPL treatment and its detailed mechanisms merit further investigations.

In conclusion, it is encouraging that our data suggested that AT-III treatment could prevent the occurrence and progression of GPL. The therapeutic effects may be associated with the inhibition of angiogenesis contributed by decreasing expression of angiogenesis-associated markers HIF-1α and VEGF-A, and by down-regulating DLL4. This study provided reliable experimental basis for the clinical treatment of GPL.

## Data Availability

The original contributions presented in the study are included in the article/Supplementary Material, further inquiries can be directed to the corresponding authors.

## References

[B1] Ambriz-Pérez D. L. Bang W. Y. Nair V. Angulo-Escalante M. A. Cisneros-Zevallos L. Heredia J. B. (2016). Protective Role of Flavonoids and Lipophilic Compounds from Jatrophaplatyphylla on the Suppression of Lipopolysaccharide (LPS)-Induced Inflammation in Macrophage Cells. J. Agric. Food Chem. 64, 1899–1909. 10.1021/acs.jafc.5b05534 26872073

[B2] Arany Z. Foo S. Y. Ma Y. Ruas J. L. Bommi-Reddy A. Girnun G. (2008). HIF-Independent Regulation of VEGF and Angiogenesis by the Transcriptional Coactivator PGC-1alpha. Nature 451, 1008–1012. 10.1038/nature06613 18288196

[B3] Bailly C. (2021). Atractylenolides, Essential Components of Atractylodes-Based Traditional Herbal Medicines: Antioxidant, Anti-Inflammatory and Anticancer Properties-Science Direct. Eur. J. Pharmacol. 891, 173735. 10.1016/j.ejphar.2020.173735 33220271

[B4] Benedito R. Roca C. Sörensen I. Adams S. Gossler A. Fruttiger M. (2009). The Notch Ligands Dll4 and Jagged1 Have Opposing Effects on Angiogenesis. Cell 137, 1124–1135. 10.1016/j.cell.2009.03.025 19524514

[B5] Du X. Cheng Z. Wang Y. H. Guo Z. H. Zhang S. Q. Hu J. K. (2014). Role of Notch Signaling Pathway in Gastric Cancer: A Meta-Analysis of the Literature. World J. Gastroenterol. 20, 9191–9199. 10.3748/wjg.v20.i27.9191 25083094 PMC4112896

[B6] Folkman J. (1971). Tumor Angiogenesis: Therapeutic Implications. N. Engl. J. Med. 285, 1182–1186. 10.1056/NEJM197111182852108 4938153

[B7] Folkman J. (2002). Role of Angiogenesis in Tumor Growth and Metastasis. Semin. Oncol. 29, 15–18. 10.1053/sonc.2002.37263 12516034

[B8] Fu J. D. Yao J. J. Wang H. Cui W. G. Leng J. Ding L. Y. (2019). Effects of EGCG on Proliferation and Apoptosis of Gastric Cancer SGC7901 Cells via Down-Regulation of HIF-1α and VEGF Under a Hypoxic State. Eur. Rev. Med. Pharmacol. Sci. 23, 155–161. 10.26355/eurrev_201901_16759 30657557

[B9] Hoang L. S. Tran M. H. Lee J. S. Ngo Q. M. Woo M. H. Min B. S. (2012). Inflammatory Inhibitory Activity of Sesquiterpenoids from Atractylodes Macrocephala Rhizomes. Chem. Pharm. Bull. (Tokyo) 64, 507–511. 10.1248/cpb.c15-00805 27150484

[B10] Hoeben A. Landuyt B. Highley M. S. Wildiers H. Van Oosterom A. T. De Bruijn E. A. (2004). Vascular Endothelial Growth Factor and Angiogenesis. Pharmacol. Rev. 56, 549–580. 10.1124/pr.56.4.3 15602010

[B11] Huai B. Ding J. (2020). Atractylenolide III Attenuates Bleomycin-Induced Experimental Pulmonary Fibrosis and Oxidative Stress in Rat Model via Nrf2/NQO1/HO-1 Pathway Activation. Immunopharmacol. Immunotoxicol. 42, 436–444. 10.1080/08923973.2020.1806871 32762376

[B12] Huang B. Jin G. Qu C. Ma H. Ding C. Zhang Y. (2019). Elevated Expression of NOTCH1 Associates with Lymph Node Metastasis of Gastric Cancer and Knock-Down of NOTCH1 Attenuates Tumor Cell Progression. Med. Sci. Monit. 25, 9939–9948. 10.12659/MSM.918703 31874951 PMC6944039

[B13] Huang L. Wu R. L. Xu A. M. (2015). Epithelial-Mesenchymal Transition in Gastric Cancer. Am. J. Transl. Res. 7, 2141–2158. 26807164 PMC4697696

[B14] Huang Q. B. Ma X. Zhang X. Liu S. W. Ai Q. Shi T. P. (2013). Down-Regulated miR-30a in Clear Cell Renal Cell Carcinoma Correlated with Tumor Hematogenous Metastasis by Targeting Angiogenesis-Specific DLL4. Plos One 8, e67294. 10.1371/journal.pone.0067294 23826258 PMC3694928

[B15] Ishigami S. Arigami T. Uenosono Y. Okumura H. Kurahara H. Uchikado Y. (2013). Clinical Implications of DLL4 Expression in Gastric Cancer. J. Exp. Clin. Cancer Res. 32, 46. 10.1186/1756-9966-32-46 23898884 PMC3751047

[B16] Ji Y. Kang Z. Kang N. Zhao Y. Guo Q. Chen Y. (2019). Atractylenolide III Enhances the Anti-Neoplastic Efficacy of Docetaxel in Gastric Cancer Cell by Inhibiting Fibroblast Growth Factor Receptors 1, -2, and -4 Expression. J. Environ. Pathol. Toxicol. Oncol. 38, 217–227. 10.1615/JEnvironPatholToxicolOncol.2019029196 31679309

[B17] Kangsamaksin T. Tattersall I. W. Kitajewski J. (2014). Notch Functions in Developmental and Tumour Angiogenesis by Diverse Mechanisms. Biochem. Soc. Trans. 42, 1563–1568. 10.1042/BST20140233 25399571

[B18] Korpanty G. Smyth E. Carney D. N. (2011). Update on Anti-Angiogenic Therapy in Non-Small Cell Lung Cancer: Are We Making Progress? J. Thorac. Dis. 3, 19–29. 10.3978/j.issn.2072-1439.2010.11.11 22263059 PMC3256499

[B19] Leyva-López N. Nair V. Bang W. Y. Cisneros-Zevallos L. Heredia J. B. (2016). Protective Role of Terpenes and Polyphenols from Three Species of Oregano (Lippia Graveolens, Lippia Palmeri and Hedeoma Patens) on the Suppression of Lipopolysaccharide-Induced Inflammation in RAW 264.7 Macrophage Cells. J. Ethnopharmacol. 187, 302–312. 10.1016/j.jep.2016.04.051 27131433

[B20] Li J. L. Sainson R. C. Oon C. E. Turley H. Leek R. Sheldon H. (2011). DLL4-Notch Signaling Mediates Tumor Resistance to Anti-VEGF Therapy *In Vivo* . Cancer Res. 71, 6073–6083. 10.1158/0008-5472.CAN-11-1704 21803743

[B21] Liu Y. R. Guan Y. Y. Luan X. Lu Q. Wang C. Liu H. J. (2015). Delta-Like Ligand 4-Targeted Nanomedicine for Antiangiogenic Cancer Therapy. Biomaterials 42, 161–171. 10.1016/j.biomaterials.2014.11.039 25542804

[B22] Malik T. H. Sayahan M. Y. Al Ahmed H. A. Hong X. (2017). Gastric Intestinal Metaplasia: An Intermediate Precancerous Lesion in the Cascade of Gastric Carcinogenesis. J. Coll. Physicians Surg. Pak 27, 166–172. 28406776

[B23] McKeage M. J. Kotasek D. Markman B. Hidalgo M. Millward M. J. Jameson M. B. (2018). Phase IB Trial of the Anti-Cancer Stem Cell DLL4-Binding Agent Demcizumab with Pemetrexed and Carboplatin as First-Line Treatment of Metastatic Non-Squamous NSCLC. Target Oncol. 13, 89–98. 10.1007/s11523-017-0543-0 29188408

[B24] Miao Z. F. Xu H. Xu H. M. Wang Z. N. Zhao T. T. Song Y. X. (2017). DLL4 Overexpression Increases Gastric Cancer Stem/Progenitor Cell Self-Renewal Ability and Correlates with Poor Clinical Outcome via Notch-1 Signaling Pathway Activation. Cancer Med. 6, 245–257. 10.1002/cam4.962 27891816 PMC5269703

[B25] Nienhüser H. Schmidt T. (2017). Angiogenesis and Anti-Angiogenic Therapy in Gastric Cancer. Int. J. Mol. Sci. 19, 43. 10.3390/ijms19010043 PMC5795993 29295534

[B26] Pimentel-Nunes P. Libânio D. Marcos-Pinto R. Areia M. Leja M. Esposito G. (2019). Management of Epithelial Precancerous Conditions and Lesions in the stomach (MAPS II): European Society of Gastrointestinal Endoscopy (ESGE), European Helicobacter and Microbiota Study Group (EHMSG), European Society of Pathology (ESP), and Sociedade Portuguesa de Endoscopia Digestiva (SPED) Guideline Update 2019. Endoscopy 51, 365–388. 10.1055/a-0859-1883 30841008

[B27] Qian C. Liu F. Ye B. Zhang X. Liang Y. Yao J. (2015). Notch4 Promotes Gastric Cancer Growth Through Activation of Wnt1/β-Catenin Signaling. Mol. Cell Biochem. 401, 165–174. 10.1007/s11010-014-2304-z 25511451

[B28] Rey S. Schito L. Wouters B. G. Eliasof S. Kerbel R. S. (2017). Targeting Hypoxia-Inducible Factors for Antiangiogenic Cancer Therapy. Trends Cancer 3, 529–541. 10.1016/j.trecan.2017.05.002 28718406

[B29] Riddell R. H. (1995). Grading of Dysplasia. Eur. J. Cancer 31A, 1169–1170. 10.1016/0959-8049(95)00134-5 7577014

[B30] Sainson R. C. Harris A. L. (2008). Regulation of Angiogenesis by Homotypic and Heterotypic Notch Signalling in Endothelial Cells and Pericytes: From Basic Research to Potential Therapies. Angiogenesis 11, 41–51. 10.1007/s10456-008-9098-0 18256896

[B31] Saito T. Inokuchi K. Takayama S. Sugimura T. (1970). Sequential Morphological Changes in N-Methyl-N'-Nitro-N-Nitrosoguanidine Carcinogenesis in the Glandular Stomach of Rats. J. Natl. Cancer Inst. 44, 769–783. 11515044

[B32] Schadler K. L. Zweidler-McKay P. A. Guan H. Kleinerman E. S. (2010). Delta-Like Ligand 4 Plays a Critical Role in Pericyte/Vascular Smooth Muscle Cell Formation During Vasculogenesis and Tumor Vessel Expansion in Ewing's Sarcoma. Clin. Cancer Res. 16, 848–856. 10.1158/1078-0432.CCR-09-1299 20103680 PMC2818539

[B33] Sheng L. Li J. Li N. Gong L. Liu L. Zhang Q. (2021). Atractylenolide III Predisposes miR-195-5p/FGFR1 Signaling axis to Exert Tumor-Suppressive Functions in Liver Cancer. J. Food Biochem. 45, e13582. 10.1111/jfbc.13582 33768570

[B34] Song H. Ekheden I. G. Zheng Z. Ericsson J. Nyrén O. Ye W. (2015). Incidence of Gastric Cancer Among Patients with Gastric Precancerous Lesions: Observational Cohort Study in a Low Risk Western Population. BMJ 351, h3867. 10.1136/bmj.h3867 26215280 PMC4516137

[B35] Tatematsu M. Aoki T. Inoue T. Mutai M. Furihata C. Ito N. (1988). Coefficient Induction of Pepsinogen 1-Decreased Pyloric Glands and Gastric Cancers in Five Different Strains of Rats Treated with N-Methyl-N'-Nitro-N-Nitrosoguanidine. Carcinogenesis 9, 495–498. 10.1093/carcin/9.3.495 3345588

[B36] Tirpe A. A. Gulei D. Ciortea S. M. Crivii C. Berindan-Neagoe I. (2019). Hypoxia: Overview on Hypoxia-Mediated Mechanisms with a Focus on the Role of HIF Genes. Int. J. Mol. Sci. 20, 6140. 10.3390/ijms20246140 PMC6941045 31817513

[B37] Tytgat G. N. (1991). The Sydney System: Endoscopic Division. Endoscopic Appearances in Gastritis/Duodenitis. J. Gastroenterol. Hepatol. 6, 223–234. 10.1111/j.1440-1746.1991.tb01469.x 1912432

[B38] Vermeulen P. B. Gasparini G. Fox S. B. Toi M. Martin L. McCulloch P. (1996). Quantification of Angiogenesis in Solid Human Tumours: An International Consensus on the Methodology and Criteria of Evaluation. Eur. J. Cancer 32A, 2474–2484. 10.1016/s0959-8049(96)00379-6 9059336

[B39] Villarreal-García D. Nair V. Cisneros-Zevallos L. Jacobo-Velázquez D. A. (2016). Plants as Biofactories: Postharvest Stress-Induced Accumulation of Phenolic Compounds and Glucosinolates in Broccoli Subjected to Wounding Stress and Exogenous Phytohormones. Front. Plant Sci. 7, 45. 10.3389/fpls.2016.00045 26904036 PMC4748037

[B40] Wang K. T. Chen L. G. Wu C. H. Chang C. C. Wang C. C. (2010). Gastroprotective Activity of Atractylenolide III from Atractylodes Ovata on Ethanol-Induced Gastric Ulcer *In Vitro* and *In Vivo* . J. Pharm. Pharmacol. 62, 381–388. 10.1211/jpp.62.03.0014 20487223

[B41] Wang S. Cai R. Ma J. Liu T. Ke X. Lu H. (2015). The Natural Compound Codonolactone Impairs Tumor Induced Angiogenesis by Downregulating BMP Signaling in Endothelial Cells. Phytomedicine 22, 1017–1026. 10.1016/j.phymed.2015.07.009 26407944

[B42] Wei P. Han T. Xin W. B. Zhang X. G. Zhang Q. Y. Jia M. (2011). Comparative Research of Chemical Constituents and Bioactivities Between Petroleum Ether Extracts of the Aerial Part and the Rhizome of Atractylodes Macrocephala. Med. Chem. Res. 20, 146–151. 10.1007/s00044-010-9311-8

[B43] Yen W. C. Fischer M. M. Axelrod F. Bond C. Cain J. Cancilla B. (2015). Targeting Notch Signaling with a Notch2/Notch3 Antagonist (Tarextumab) Inhibits Tumor Growth and Decreases Tumor-Initiating Cell Frequency. Clin. Cancer Res. 21, 2084–2095. 10.1158/1078-0432.CCR-14-2808 25934888

[B44] Zhao W. X. Liu Z. F. Li X. L. Li Z. (2019). Correlations of Serum Homocysteine, VEGF and Gastrin 17 with Gastric Cancer and Precancerous Lesions. Eur. Rev. Med. Pharmacol. Sci. 23, 4192–4198. 10.26355/eurrev_201905_17922 31173290

[B45] Zheng L. Shao Z. D. Wang Z. C. Fu C. X. (2012). Isolation and Characterization of Polymorphic Microsatellite Markers from the Chinese Medicinal Herb Atractylodes Macrocephala (Asteraceae). Int. J. Mol. Sci. 13, 16046–16052. 10.3390/ijms131216046 23443109 PMC3546677

